# P-877. Trends and Indications for Dalbavancin Use in a Large U.S. Healthcare Network: A Retrospective Analysis from 2016-2024

**DOI:** 10.1093/ofid/ofaf695.1085

**Published:** 2026-01-11

**Authors:** Chandan K Dash, Matthew A Moffa, Thomas L Walsh, Sarah Kanell, Nathan Hammerle, Dustin R Carr, Derek N Bremmer, Tamara Trienski, George Bchech, Alexander Tarr, Carley Buchanan, Shenjun Zhu, Nathan R Shively

**Affiliations:** Medical Graduate, PIttsburgh, Pennsylvania; Allegheny Health Network, Pittsburgh, Pennsylvania; Allegheny Health Network, Pittsburgh, Pennsylvania; Allegheny Health Network, Pittsburgh, Pennsylvania; Allegheny Health Network, Pittsburgh, Pennsylvania; Allegheny General Hospital, Pittsburgh, Pennsylvania; Allegheny General Hospital, Pittsburgh, Pennsylvania; Allegheny General Hospital, Pittsburgh, Pennsylvania; Allegheny Health Network, Pittsburgh, Pennsylvania; Allegheny Health Network, Pittsburgh, Pennsylvania; Allegheny Health Network, Pittsburgh, Pennsylvania; Allegheny Health Network, Pittsburgh, Pennsylvania; Allegheny Health Network, Pittsburgh, Pennsylvania

## Abstract

**Background:**

Dalbavancin, a long-acting lipoglycopeptide antibiotic, was approved by the FDA in 2014 for the treatment of adult patients with acute bacterial skin and skin structure infections. There are limited published data evaluating the trends and indications for use of dalbavancin in the United States.

**Figure 1:**
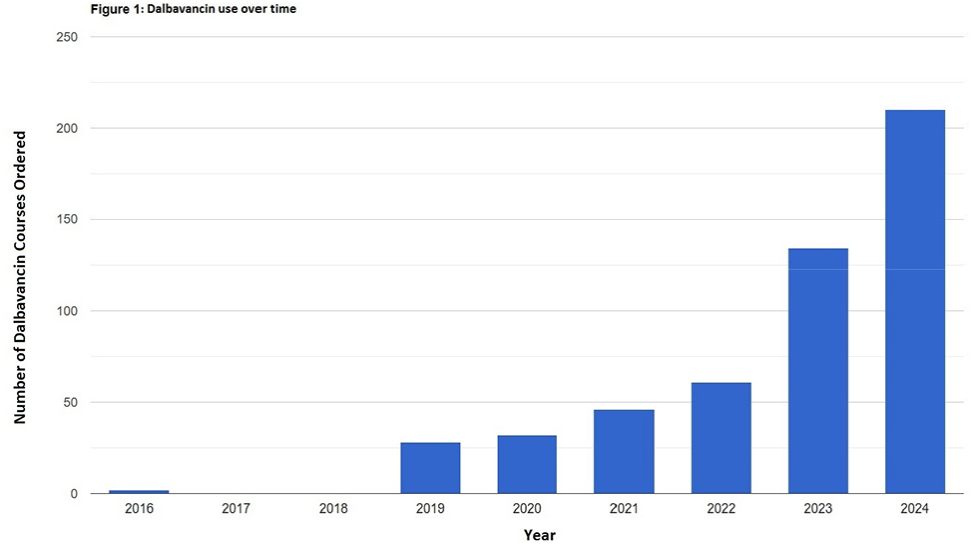
Dalbavancin use over time

**Table 1:**
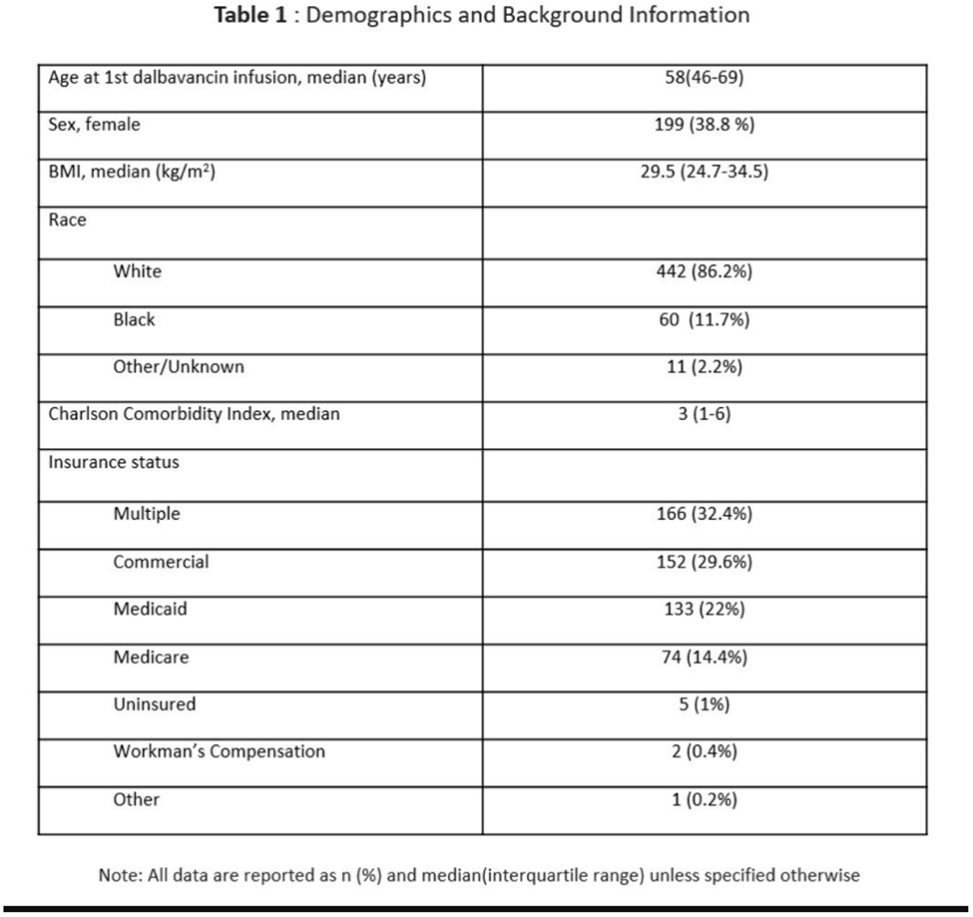
Demographics and Background Information

**Methods:**

We identified all orders for dalbavancin in our healthcare system from 01/01/2016 to 12/31/2024, with a treatment course considered one or more planned infusions for the same infection. We collected demographic data, insurance status, and number of dalbavancin infusions received. The indication for dalbavancin was manually collected by a chart review in a subset of 270 patients.

**FIgure 2:**
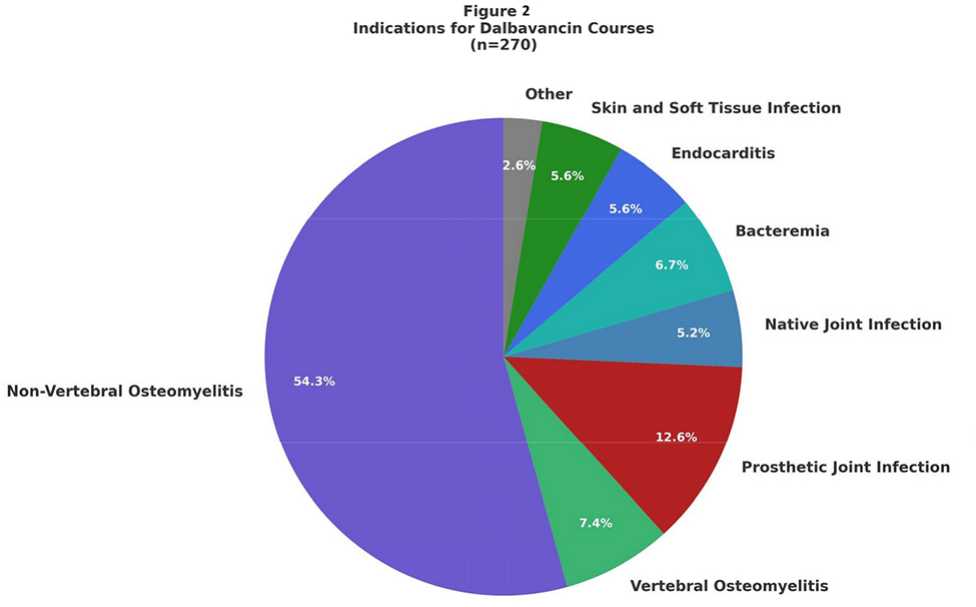
Indications for dalbavancin courses

**Figure 3:**
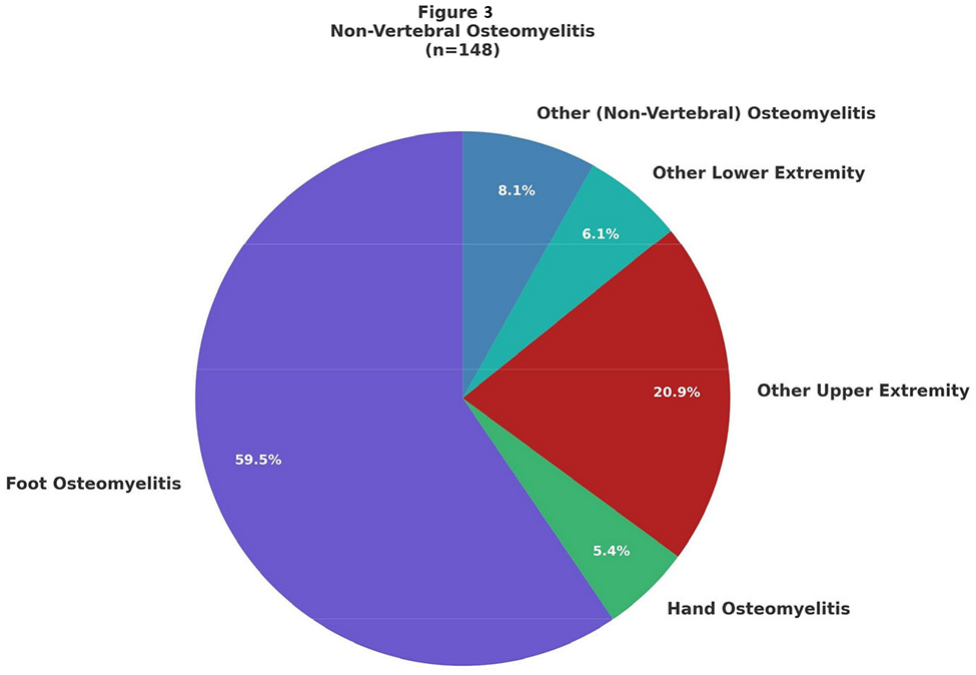
Non-vertebral osteomyelitis

**Results:**

There were 513 dalbavancin treatment courses prescribed with an increasing trend annually, from 2 courses in 2016 to 210 courses in 2024 (Fig. 1). Most treatment courses consisted of 2 infusions (80.1%), with 19.1% of courses consisting of one infusion and 0.8% of courses with 3 or more infusions. Demographic data can be found in Table 1 and include age (median: 58 years), gender, race, ethnicity, body mass index (BMI) (median 29.5 kg/m2). Insurance data (most common insurance: multiple) and Charlson comorbidity index (median 3) data can be found in Table 1. Among reviewed cases, the most common indication for dalbavancin was osteomyelitis (61.8%), followed by prosthetic joint infection (12.6%), bacteremia (6.7%), endocarditis (5.6%), skin and soft tissue infection (5.6%), and native joint infection (5.2%) (Fig. 2 and 3). 58 (40.8%) patients with osteomyelitis had hardware related infections. Out of 21 patients with vertebral osteomyelitis, 9 (42.9%) patients had epidural abscesses.

**Conclusion:**

We observed an increasing trend in the number of dalbavancin courses given from 2016-2024. A large number of these courses were ordered for off-label indications such as osteomyelitis, joint infections, bacteremia and infective endocarditis. Further research is needed to describe the effectiveness of dalbavancin for these indications.

**Disclosures:**

Dustin R. Carr, PharmD, BCPS, BCIDP, Merck: Honoraria

